# Uncovering the PIDDosome and caspase-2 as regulators of organogenesis and cellular differentiation

**DOI:** 10.1038/s41418-020-0556-6

**Published:** 2020-05-15

**Authors:** Valentina C. Sladky, Andreas Villunger

**Affiliations:** 1 Division of Developmental Immunology, Biocenter, Medical University of Innsbruck, Innsbruck, Austria; 2 Ludwig Boltzmann Institute for Rare and Undiagnosed Diseases, 1090 Vienna, Austria; 3 CeMM Research Center for Molecular Medicine of the Austrian Academy of Sciences, 1090 Vienna, Austria

**Keywords:** Cell biology, Genetics

## Abstract

The PIDDosome is a multiprotein complex that drives activation of caspase-2, an endopeptidase originally implicated in apoptosis. Yet, unlike other caspases involved in cell death and inflammation, caspase-2 seems to exert additional versatile functions unrelated to cell death. These emerging roles range from control of transcription factor activity to ploidy surveillance. Thus, caspase-2 and the PIDDosome act as a critical regulatory unit controlling cellular differentiation processes during organogenesis and regeneration. These newly established functions of the PIDDosome and its downstream effector render its components attractive targets for drug-development aiming to prevent fatty liver diseases, neurodegenerative disorders or osteoporosis.

## Graphical abstract

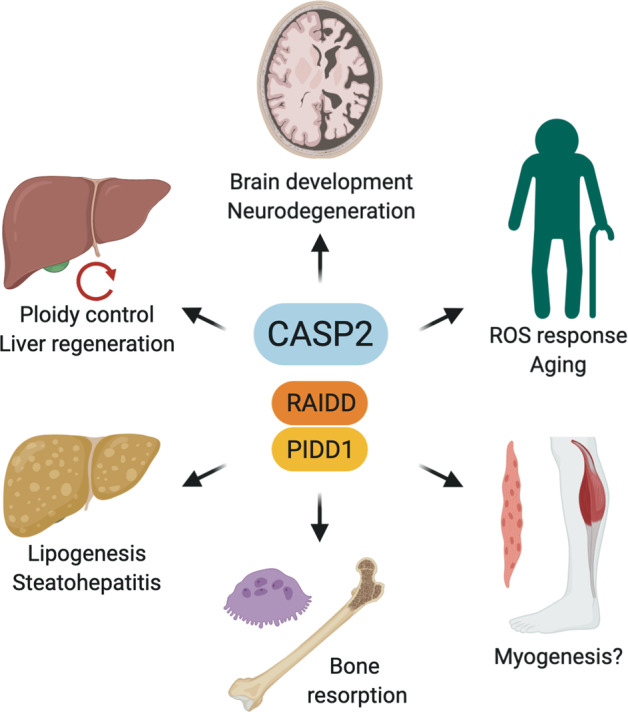

## Facts

- Caspase-2 and the PIDDosome are involved in the differentiation of various cell types.
- Caspase-2, PIDD1 and RAIDD have roles outside the PIDDosome.
- Caspase-2 has nonapoptotic functions.

## Open questions

- How is the PIDDosome activated by extra centrosomes in polyploid cell types?
- Are there alternative activation platforms for caspase-2?
- How is caspase-2 activated in neurodevelopment and regeneration?
- Is the dual adapter RAIDD part of other protein complexes?

## Introduction

Caspases are a family of cysteine proteases mainly known for their roles in apoptosis and inflammation. They are grouped according to structure and function into initiators and executioners. Initiator caspases-8 and -9 are activated on high molecular weight complexes facilitating proximity-induced dimerization and autoprocessing. During apoptosis, initiator caspases proteolytically activate the effector caspases-3 and -7, which finally execute cell death [1]. Although mainly known for their function in apoptosis, these proteases can also perform other tasks [2]. Apoptotic and nonapoptotic caspase activity plays a crucial role during development and differentiation in various tissues and cell types such as macrophages, osteoblasts and neural stem cells [3]. So far, mainly the well-studied classical apoptotic initiator caspases-8 and -9 as well as the effector caspases-3 and -7 have been investigated in this context [3]. However, cumulative evidence also implicates caspase-2 as an important driver of cell maturation and differentiation processes.

**Fig. 1:**
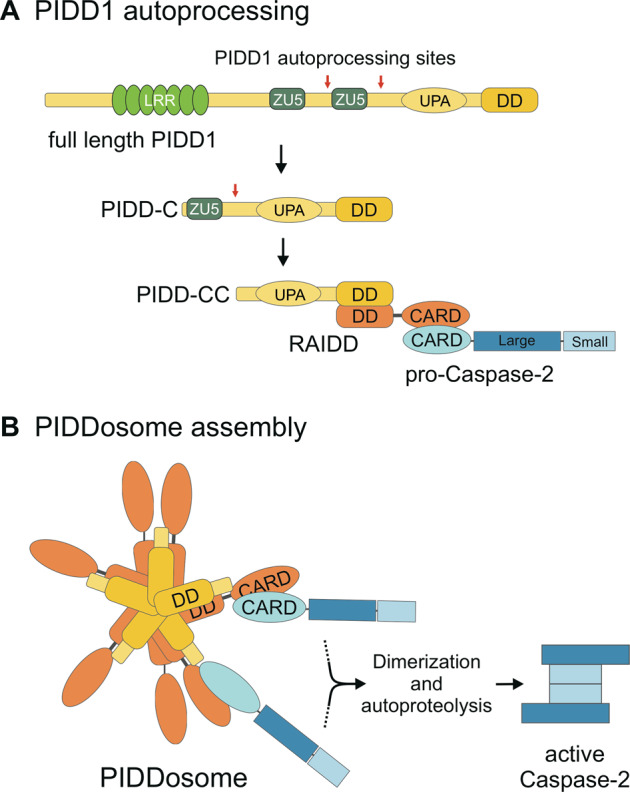
PIDD1 autoprocessing and caspase-2 activation. **a** Full-length PIDD1 undergoes autoprocessing at specific residues S446 and S588 (red arrows) to generate two C-terminal fragments with distinct functions, PIDD-C and PIDD-CC. The former has been implicated in NFκB signaling while the latter interacts with the death domain (DD) of RAIDD to form the PIDDosome. The caspase activation and recruitment domain (CARD) of RAIDD interacts with procaspase-2. **b** The core of the PIDDosome complex is formed by 5:5 PIDD-CC:RAIDD molecules interact via the DDs, and two additional RAIDD entities are placed on top protruding in diametric orientation (based on the protein database structure 2OF5). Binding of RAIDD to procaspase-2 promotes proximity-induced dimerization and autocleavage of caspase-2, critical for its activation.

Caspase-2 shares structural similarities with the apoptotic initiator caspase-9, as it contains a caspase activation and recruitment domain (CARD) and its activation requires dimerization and subsequent autoprocessing [4]. In analogy to caspase-9 and the apoptosome, the multimeric protein complex implicated in caspase-2 activation was dubbed the “PIDDosome” [5]. It consists of the C-terminal fragment of PIDD1 (p53-induced death domain protein 1) and RAIDD (receptor-interacting protein (RIP)-associated ICH-1/CED-3 homologous protein with a death domain; alias CRADD), in a 5:5 stoichiometry with two additional RAIDD molecules on top of the core structure [5–7]. The core of the complex is formed by PIDD-CC, a fragment of PIDD1 generated by autoprocessing (Fig. 1a, reviewed in [8, 9]). RAIDD has a dual adapter function. It contains a C-terminal death domain (DD), which binds the DD of PIDD1 while the N-terminus carries the CARD, which mediates the homotypic interaction with procaspase-2 [5, 10]. Binding of procaspase-2 to the complex brings the caspase-2 monomers in close proximity, which induces dimerization, autocatalytic cleavage and thus activation (Fig. 1b) [11].

The PIDDosome was initially described to activate caspase-2 upon genotoxic stress to induce apoptosis [5]. However, caspase-2 can be activated independently of the PIDDosome, and also PIDD1 has been reported to form alternative protein complexes, which, among other tasks, can drive NF-κB signaling or assist trans-lesion DNA synthesis [12–14] (reviewed in [8, 9]). Aside from DNA damage several other cues for the activation of caspase-2 within or outside the PIDDosome have been suggested. The set of triggers is very diverse and includes heat shock, interference with the cytoskeleton, or the accumulation of β-amyloids [15–17] (reviewed in [18]). Most importantly, caspase-2 and the PIDDosome can function in a “polyploidy checkpoint” [19]. Triggered by the presence of supernumerary centrosomes, mostly occurring after failed cell division (cytokinesis), the PIDDosome activates caspase-2, which proteolytically inactivates MDM2, thus inducing a p53 response. Recent reports also implicate caspase-2 in aneuploidy surveillance in cancer, although the mechanistic details are still unclear [16–18].

Characterization of the mutant mice lacking *Casp2*, *Pidd1*, or *Raidd* was initially unrevealing, especially because many of the proposed functions based on cell line studies could not be recapitulated in vivo. Mice lacking individual PIDDosome components are all viable, born in Mendelian ratios, and do not exhibit gross defects in development or growth nor the DNA-damage response [12, 20–22]. Yet, detailed characterization of these genotypes revealed several subtle defects and impairments. When challenged, however, the effects found in mutant mice imply versatile functions of the PIDDosome components either together or independently, under physiological and under pathological conditions. Of note, most of the early studies focused on a cell-death-related role of these proteins, and thus potential nonapoptotic functions might have been overlooked. The roles of the PIDDosome components in cell death and tumorigenesis have been extensively reviewed before [18, 23, 24]. Here, we aim to discuss where, when, and how caspase-2 and the PIDDosome shape cell maturation and differentiation processes in diverse tissues, as well as their emerging roles in tissue homeostasis and regeneration.

## From oocyte death to old age

Initially, caspase-2 was deemed a strong candidate for driving apoptosis during embryonic development, as in mice it is highly expressed between E8 and E16. Yet, in contrast to loss of caspase-9, mice devoid of caspase-2 come to term and develop normally [20, 25]. Specific cell types, however, were found to be less sensitive to apoptotic triggers in the absence of caspase-2. Most prominently, caspase-2 null oocytes are highly resistant to cell death upon doxorubicin exposure. Accordingly, female mutant mice present with excess oocytes confirming that, indeed, caspase-2 controls oocyte numbers [20]. Interestingly, also *Xenopus* oocytes depend on caspase-2 to undergo apoptosis upon nutrient depletion. The metabolic flux through the pentose phosphate pathway maintains an inhibitory phosphorylation by CaMKII on caspase-2, which mediates its binding to 14-3-3ζ. Nutrient exhaustion, however, promotes release of 14-3-3ζ from caspase-2 allowing subsequent dephosphorylation by PP1. This, in turn, allows interaction with RAIDD and thus activation, as shown in oocyte extracts [26, 27]. Moreover, the interaction with 14-3-3ζ was recently reported to block both the NLS (nuclear localization signal) and the dimerization interface of caspase-2 [28, 29]. Whether the activation of caspase-2 in *Xenopus* oocytes also involves PIDD1 or if RAIDD binds to an alternative interactor in this context has not been studied to date. In mouse oocytes, however, a role for PIDD1 can be excluded as knockout mice have regular numbers of oocytes that are normally sensitive to doxorubicin treatment [21]. To date, this has not been investigated in *Raidd-*deficient mice.

Studies on caspase-2-deficient mice have revealed several phenotypes which can be summarized as signs of premature aging [30–34]. Most obviously, the average live-span of *Casp2*^*−/−*^ mice is about 4 months shorter, the bone volume is reduced, and hematopoiesis is skewed towards the myeloid compartment compared to the wild-type counterparts [31, 32, 34]. Another strong indication of premature aging was described in caspase-2-deficient mouse embryonic fibroblasts (MEFs) and lymphoma cells. These cells exhibit weaker telomere staining, indicative of telomere shortening [35]. The major cause of early onset of aging in the absence of caspase-2 seems to be increased oxidative stress. Caspase-2 was suggested to control the levels of reactive oxygen species (ROS) via FoxO transcription factors, as their expression levels correlate with those of caspase-2. As tested in the aged mouse liver, caspase-2 deficiency results in reduced mRNA expression of FoxO1 and FoxO3a as well as their transcriptional targets, such as the antioxidant enzymes catalase or SOD2 (superoxide dismutase 2) [31]. Although the mechanistic relation is still unclear, these data point out that caspase-2, directly or indirectly, affects the oxidative stress response. Accordingly, *Casp2*^*−/−*^ mice exhibit higher numbers of senescent cells and increased DNA damage in the liver and the bone marrow [31, 32].

Furthermore, proteomics and metabolomics analyses comparing young and aged wild-type and caspase-2-deficient livers or serum revealed deregulation of several pathways in the absence of caspase-2. These include oxidative phosphorylation as well as amino acid, glucose, and fatty acid metabolism [30]. Even though the regulatory mechanisms are not understood yet, most of these alterations can be secondary consequences of elevated oxidative stress or altered cellular ploidy (see below). Together, these observations clearly highlight the contribution of caspase-2-regulated signaling in various tissues and cell types during development, regeneration and aging. Again, the roles of PIDD1 and RAIDD in controlling ROS levels or aging have not been tested.

## Liver metabolism and ploidy control

**Fig. 2:**
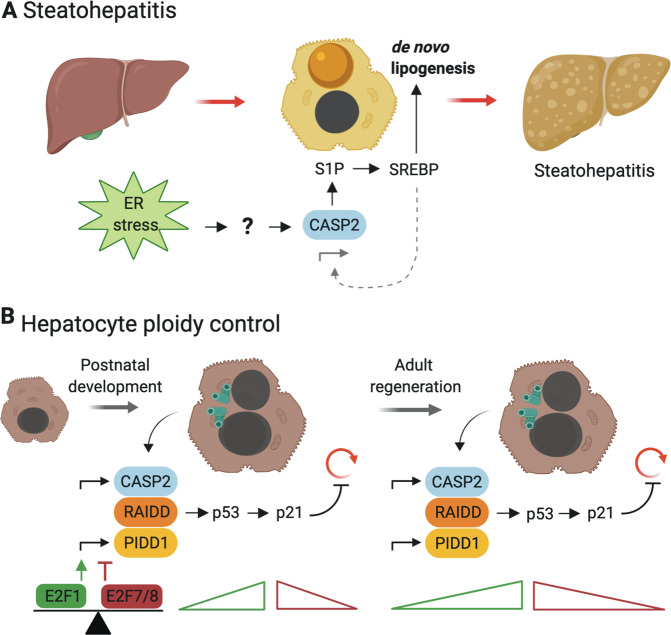
Caspase-2 controls liver metabolism and ploidy. **a** ER-stress can induce the expression of caspase-2 in hepatocytes, which then activates site 1 protease (S1P), localized at the ER, by targeted proteolysis. Active S1P cleaves and activates the SREBP transcription factors (sterol regulatory element-binding protein) that control gene expression programs involved in lipogenesis, and also targets the caspase-2 promoter itself in a feed forward loop. **b** Upon cytokinesis failure, proliferating hepatocytes in the developing liver engage the PIDDosome for caspase-2 activation to promote p53 induction and p21-dependent cell cycle arrest. Expression of caspase-2 and PIDD1 is under the control of E2F family proteins and thus linked to proliferation. Both PIDDosome components are barely expressed in adult hepatocytes, but are reactivated in an E2F-dependent manner to avoid hyper-polyploidization during liver regeneration.

Interestingly, cumulative evidence suggests a pivotal role for caspase-2 in lipid metabolism (Fig. 2a). In addition to the above-mentioned findings using Omics approaches, caspase-2 was shown to promote de novo lipogenesis in the liver [33, 36]. In hepatocytes, caspase-2 localizes to the ER where it cleaves and activates S1P (site 1 protease). S1P, in turn, can process and thus release activated SREBP1 and SREBP2 (sterol regulatory element-binding protein) [36], the main regulators of fatty acid and cholesterol synthesis, respectively [37]. A recent study using the human hepatocyte cell line Huh7 suggests that phosphorylation of caspase-2 at T180, possibly by p38 MAPK, promotes SREBP activation [38]. Caspase-2-controlled lipogenesis occurs in response to TNF but also ER-stress, a condition reported before to involve caspase-2 [39]. If and how ER-stress impacts on the regulation of caspase-2 levels has been a matter of debate, given contradicting findings in MEF or leukocytes exposed to brefeldin A, thapsigargin or tunicamycin [39, 40]. Clearly, though, in mouse hepatocytes that do not show appreciable caspase-2 protein levels in steady state, overexpression of urokinase, a strong driver of ER-stress, leads to a concomitant upregulation of caspase-2 [36]. Interestingly, the caspase-2 promoter carries several sterol response elements [41]. Hence, SREBP activation might trigger a positive feedback loop to further increase lipid synthesis by upregulating caspase-2 expression. In line with these findings on lipid metabolism, caspase-2 mice have lower body weight, reduced fat deposition, and smaller adipocytes, and are largely protected against hepatic steatosis [33, 36, 42, 43]. This interrelation of caspase-2 and liver steatosis was first proposed based on high caspase-2 levels in patients with severe nonalcoholic steatohepatitis (NASH) [43]. To further investigate caspase-2 in this context, various diets in combination with mouse models for NAFLD (nonalcoholic fatty liver disease) and NASH were used: high-fat diet (HFD), methionine choline-deficient diet (MCD), obese and diabetic mice, as well as *MUP-uPA* transgenic mice. In the latter, exogenous expression of urokinase induces ER-stress, which cooperates with HFD to model NASH [44]. Strikingly, all studies revealed that lack of caspase-2 is highly protective against fatty liver diseases, independent of the model used [36, 42, 43]. Initially, this effect was ascribed to a proapoptotic function of caspase-2 in lipotoxicity-induced cell death. However, the MCD diet used in this study is highly hepatotoxic by itself [42, 43]. In contrast, Kim et al. [36] suggest that the major contribution of caspase-2 in NAFLD and NASH is driving lipogenesis rather than lipoapoptosis. The authors utilized the HFD *MUP-uPA* model, which is presumed to be a better surrogate for the human disease condition [44].

Taken together, these studies show that caspase-2 is transcriptionally upregulated in these pathologic conditions and promotes progression of fatty liver diseases (Fig. 2a). Yet, the mode and location of caspase-2 activation in this setting still has to be resolved, as the active protease needs to act in the ER for S1P processing. According to previous studies, exogenously expressed caspase-2 is preferentially found in the nucleus [45, 46], while endogenous caspase-2 has been reported to localize to the nucleus, cytoplasm, and golgi [47] in steady state. Caspase-2 activity was also detected in the nucleolus using a bi-fluorescent reporter system, which utilizes a split fluorophore coupled to the caspase-2-CARD to indicate the site of dimerization [48]. The site(s) of endogenous caspase-2 activation and how trafficking to the ER is achieved have to be further investigated. Remarkably, mice deficient for either PIDD1 or RAIDD stay also lean until old age and rarely display liver steatosis, a condition frequently observed in aged male wild-type mice (own unpublished data, Di Donato et al., 2016). These highly similar phenotypes strongly suggest that the PIDDosome is involved in ER-stress-induced caspase-2 activation to promote de novo lipogenesis.

Aside from its function in liver metabolism, we recently reported a pivotal role for caspase-2 in liver polyploidization (Fig. 2b). During murine postnatal liver development induced by weaning, proliferating hepatocytes perform incomplete cell division and become polyploid [49, 50]. This process of polyploidization is regulated by insulin signaling as well as by E2F transcription factors [51–53]. Although the reason for liver polyploidy is not fully understood, it was deemed essential to protect against tumorigenesis as increased genome copy number can buffer against genotoxic stress [54–56]. Hepatocyte polyploidization is a highly regulated process and we recently identified that caspase-2 and the PIDDosome define its upper limit [19, 57]. In polyploid hepatocytes, the PIDDosome is triggered presumably by accumulated centrosomes and activates caspase-2, which in turn stabilizes p53 leading to p21 induction to halt proliferation in a polyploid state [19, 57]. Moreover, the same effect was observed during liver regeneration, where loss of caspase-2, PIDD1 or RAIDD accelerates regeneration accompanied by excessive polyploidization (Fig. 2b). To control PIDDosome function in the presence of its activating cue, extra centrosomes, in polyploid hepatocytes, caspase-2 expression is tightly controlled by E2F transcription factors. Of note, E2F1 binds to the promoters of *CASP2* and *PIDD1*. It not only coregulates their expression in proliferative phases during early postnatal development but also during regeneration. E2F7 and E2F8, themselves E2F1 targets that compete for the same binding sites on DNA, act as repressors of both genes. Thus, expression and activation is restricted to proliferative phases but prevented in quiescent hepatocytes [57]. Here, caspase-2 activation clearly requires the PIDDosome as activating platform, and mice deficient in either PIDD1 or RAIDD fully mimic caspase-2 deletion.

In summary, in the liver, E2F-controlled caspase-2 expression is closely associated with proliferation [57]. This may provide an explanation for its upregulation seen in NASH and NAFLD that may happen in addition to SREBP-mediated transcriptional induction of caspase-2 [41], as these diseases are characterized by loss of functional parenchyma and compensatory proliferation [58]. Thus, it is possible that proliferation-driven increases in caspase-2 levels further feeds into disease progression by enhancing lipogenesis. Remarkably, expression of *RAIDD/CRADD* is entirely uncoupled from that of *CASP2* or *PIDD1*. It is constitutively expressed during all stages of liver development as well as regeneration, suggesting it may have additional functions and serve the PIDDosome only in some occasions [57]. Aside from SREBPs and E2Fs, another proliferation-associated pathway has been implicated in transcriptional regulation of *CASP2*. In colorectal cancer cell lines, the Wnt signaling component BCL9L was found to positively regulate caspase-2 mRNA and protein levels. The *CASP2* promoter region harbors a TCF4 binding site, and BCL9L cooperates with this transcription factor to induce *CASP2* expression [59]. While mRNA levels of PIDD1 are not affected [59], it has to be tested whether BCL9L and TCF4 also control the expression of *RAIDD/CRADD*.

## Differentiation processes in bone and skeletal muscle

Osteoclasts, cells of the hematopoietic lineage, are responsible for bone resorption. Together with the bone-building osteoblasts, which are of mesenchymal origin, osteoclasts maintain bone homeostasis. Osteoclast maturation from myeloid progenitors is induced by RANKL (Receptor Activator of NF-κB Ligand), a differentiation factor mainly produced by osteoblasts thus ensuring balanced bone remodeling [60]. In addition, osteoclast differentiation requires low levels of ROS [61]. In order to mature, osteoclasts undergo polyploidization, which enhances their bone resorption capacity [62]. Although polyploidy was long assumed to be a result of cell-to-cell fusion, osteoclasts were recently found to additionally perform endomitosis [63]. This was tested utilizing the FUCCI (Fluorescent Ubiquitination-based Cell Cycle Indicator) system for cell cycle tracking. The FUCCI system contains two fluorescent probes fused to degrons derived from Cdt1 and Geminin, which are present in G1-phase and S/G2/M-phase, respectively, and thus enable to discriminate the cell cycle stage [64]. Using RANKL-stimulated monocytes from FUCCI mice, the authors show that osteoclasts also increase their nuclear ploidy and proliferate without completing cytokinesis. In general, cells becoming polyploid either by endomitosis or cell fusion also accumulate extra centrosomes respective of their total DNA content, as centrosome biogenesis is tightly coupled to DNA replication [65].

**Fig. 3:**
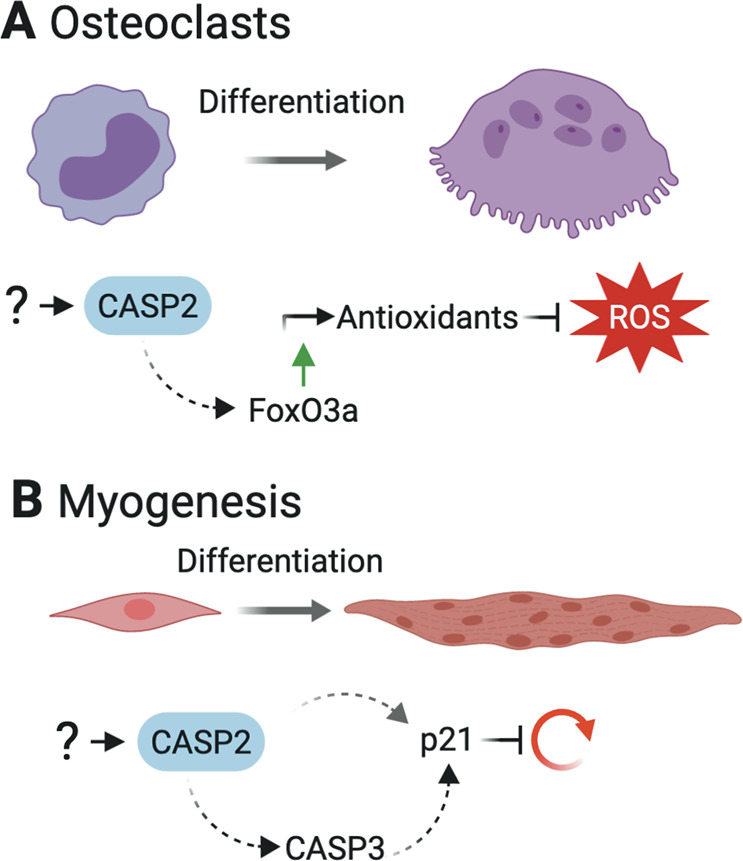
Terminal differentiation programs controlled by caspase-2. **a** Caspase-2 has been implicated in osteoclast differentiation by controlling FoxO3a transcription factor activity, which controls expression of ROS scavenging enzymes such as SOD2 and thus ROS levels that are critical for osteoclastogenesis. **b** Cell cycle exit for differentiation of myoblasts requires p21 induction, which is reportedly caspase-2-dependent. Caspase-2 may play an apical function in the activation of caspase-3 and p21 to promote myoblast differentiation.

Caspase-2 is involved in osteoclast differentiation (Fig. 3a). This was attributed to its function in regulating the transcription factor FoxO3a and thus ROS levels, an important factor promoting osteoclastogenesis [31, 66]. Even though low ROS levels are needed for differentiation, abnormally high ROS levels damage the cell, which is proposed to activate caspase-2 to mediate apoptosis [67]. Thus, in the absence of caspase-2, osteoclasts with ROS levels reaching the apoptotic threshold survive and show enhanced osteoclast activity, which was measured by TRAP (tartrate-resistant acid phosphatase) activity and cathepsin K levels, two enzymes required for bone matrix resorption [66, 67]. In accordance, caspase-2-deficient mice have higher numbers of osteoclasts and thus an imbalance in bone homeostasis [34, 66], which may cause the reduced bone mineral density and fracture strength, reflecting bone fragility and loss, in aged mice [31, 34].

The activating cue for caspase-2 during osteoclast differentiation is still unclear, and also the potential involvement of the PIDDosome as activation platform has not yet been addressed. Intriguingly, osteoclasts derived from caspase-2-deficient mice are larger and harbor more nuclei per cell. Moreover, an increase in total cell numbers and highly polyploid cells was observed in these cultures [66]. This noted increase in cell size together with uncontrolled ROS levels might cause the augmented activity of *Casp2*^*−/−*^ osteoclasts, as both factors are known to enhance the bone resorption capacity [62, 66]. Activation of caspase-2 was mostly found during the later stages of maturation when polyploidization occurs [66]. Hence, it is possible that caspase-2 is activated by the accumulation of extra centrosomes engaging the PIDDosome to regulate polyploidy and thus differentiation. Whether *Pidd1*^*−/−*^ and *Raidd*^*−/−*^ mice show the same phenotype in bone weakening has to be investigated.

Recently, a role for caspase-2 in muscle cell differentiation and fusion was proposed [68] (Fig. 3b). Formation and regeneration of skeletal muscle requires myoblasts to exit the cell cycle, differentiate, and subsequently fuse to generate multinucleated myotubes [69]. Among the wide range of signals involved, limited DNA damage caused by caspase activity has been shown to be crucial for these processes. An in vitro study using C2C12 mouse myoblasts revealed that sublethal caspase-3 activity drives CAD (caspase activated DNAase) activation, causing DNA damage without the induction of apoptosis [70]. DNA damage, in turn, triggers DNA repair signaling and the expression of the cell cycle inhibitor p21 for cell cycle exit [70–73]. How myoblasts activate caspase-3 is not fully understood, but it is clearly independent of mitochondrial apoptotic signaling [74]. Caspase-2 has been suggested to act upstream of caspase-3, as both of them show increased activity during the early stages of C2C12 myoblast differentiation in vitro [68]. Activity of caspase-2 and -3 was measured using the fluorogenic substrates Ac-VDVAD-AMC and Ac-DEVD-AMC, respectively [68]. Of note, this substrate assumed to be caspase-2-specific is also processed by caspase-3 [75]. Albeit this technical issue, caspase-2 enhances p21 expression in these cells, which was abrogated by RNAi-mediated knockdown or pharmacological inhibition of caspase-2 [68]. In summary, the authors suggest that caspase-2 has an apical function in the activation of caspase-3 and p21 and thus promotes myoblast differentiation. Although loss of caspase-2 did not prevent cell cycle exit but rather accelerated it, caspase-2 depletion clearly abrogated fusion of C2C12 myoblasts [68]. The link between caspase-2 and p21 in this setting is less clear, though. Caspase-2 can either cleave MDM2, leading to p53 and p21 induction, or promote p21 expression via the above-mentioned route of caspase-3 and CAD, which directly targets the p21 promoter to induce transcription by introducing DNA breaks [73, 76].

The upstream signal activating caspase-2 has not been directly investigated. DNA damage could be a potential trigger although most reports so far link it to an apoptotic outcome [77, 78]. On the other hand, supernumerary centrosomes accumulating in myotubes could activate caspase-2 in a PIDDosome-dependent manner [19]. Considered an additional mechanism for cell cycle exit, myotubes dissolve their centrosomes after fusion [79]. Still, there is a short time window in which extra centrosomes accumulate that could potentially activate the PIDDosome and caspase-2 during differentiation. However, possible roles of PIDD1 and RAIDD await detailed studies.

The role of p21 in myogenesis could be confirmed in vivo, as mice devoid of p21 have delayed muscle regeneration upon Bupivacaine-induced injury [72]. In contrast, mice deficient in caspase-2, PIDD1, or RAIDD were not reported to have any obvious muscular defects. Also, muscle fibers of *Casp2*^*−/−*^ mice appear normal in terms of diameter and shape under normal conditions as well as after regeneration from cardiotoxin-induced injury (own unpublished data). Furthermore, rotarod and swimming tests revealed normal motor function in caspase-2-deficient mice [80]. However, caspase-9 has also been suggested to act upstream of caspase-3 in C2C12 myoblasts and could compensate for loss of caspase-2 during development in vivo [81]. Hence, a function for caspase-2 in myogenesis cannot be excluded by the fact that these mice develop apparently normal, fully functional skeletal muscle tissue.

## Caspase-2 and the PIDDosome in neuronal development and diseases

Already the first study in *Casp2*^*−/−*^ mice suggested its involvement in neuronal cell death. Somewhat counterintuitive, Bergeron et al. [20] reported that caspase-2 delays cell death of facial motor neurons but no effect was seen in sympathetic neurons isolated from caspase-2-deficient animals upon withdrawal of NGF. This was challenged by the finding that loss of caspase-2 is indeed protective against NGF deprivation in these cells, suggesting that caspase-2 loss may be compensated for by other caspases in vivo [17, 82]. Moreover, Troy et al. [17] implicated caspase-2 in Alzheimer’s disease (AD), as it mediates Aβ-induced apoptosis in hippocampal and sympathetic neurons. The upstream activating signal as well as downstream cell death mediators remain to be determined, although the proapoptotic BCL2 family member BID has been suggested as the prime caspase-2 effector mediating  cerebral ischemia-induced apoptosis [83]. Moreover, the authors provide first evidence that the PIDDosome is responsible for direct caspase-2 activation as the PIDD-CC fragment is, at least partially, required for caspase-2 activation in hippocampal neurons of the CA1 region [83]. Somewhat contradictory though, Ribe et al. [82] used RNAi knockdown as well as neurons derived from PIDD1-deficient mice and reported that PIDD1 is dispensable for both Aβ and NGF deprivation-induced neuronal cell death. Nonetheless, RAIDD clearly is required for caspase-2 activation in the neuronal cell line PC12 as well as sympathetic and hippocampal neurons. This was confirmed as RNAi-mediated knockdown of RAIDD abrogated caspase-2 processing and activity. The latter was measured using a biotin-VAD-FMK assay, which allows to identify active caspases by labeling for affinity purification and subsequent immunoblotting [82, 84].

**Fig. 4:**
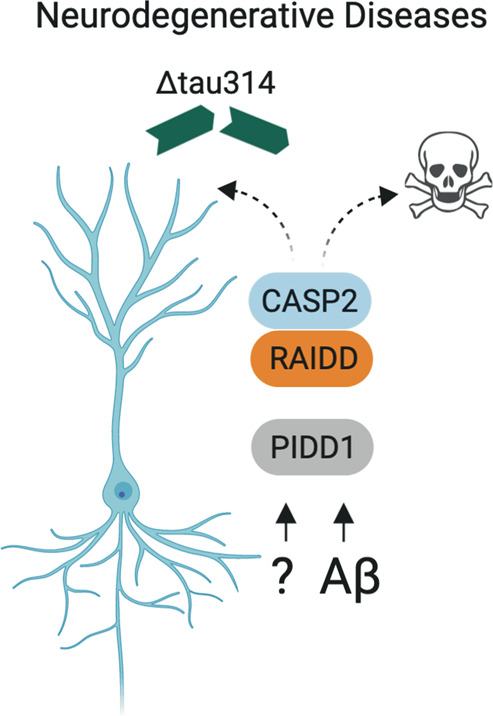
Caspase-2 in the neuronal stress response. Caspase-2 has been implicated in neurodegenerative diseases, including Alzheimer’s disease (AD), as it mediates Aβ-induced apoptosis in hippocampal and sympathetic neurons in an RAIDD-dependent manner. Caspase-2 may also contribute to AD in a nonapoptotic way by cleaving the tau protein at Asp314, producing a soluble, truncated version, which causes decreased excitatory synaptic transmission and impairs memory in diseased mice. The role of PIDD1 in either process remains uncertain.

Caspase-2 has been shown to mediate Aβ toxicity and alter dendritic spine morphology not only in neuron cultures but also in vivo, where caspase-2 deletion rescues memory loss seen in the J20 APP mouse model for AD [85]. Moreover, caspase-2 contributes to AD in a nonapoptotic way, as it cleaves the tau protein at Asp314, producing a soluble, truncated version, which can delocalize into dendritic spines. This results in decreased excitatory synaptic transmission and impairs memory in diseased mice [86]. Loss of caspase-2 was shown before to restore memory and other cognitive defects in a mouse model for Huntington’s disease, further supporting these findings [87]. Intriguingly, recent reports suggest that, indeed, caspase-2-dependent generation of Δtau314 is also responsible for the cognitive deficits seen in Lewy body disease, non-dementia Parkinson’s disease as well as Huntington’s disease [88, 89]. Strikingly, a recent study on human individuals suffering from AD found that the levels of Δtau314 correlate with cognitive impairment, and also caspase-2 protein levels were increased in these patients, confirming the findings made in mouse models [90]. In addition to cleavage of tau, caspase-2 was found to shape dendritic spines by regulating AMPA receptor internalization via proteolytic cleavage of Rictor, thus inhibiting mTORC2 signaling. This reduces synaptic strength and allows removal of synapses. Hence, lack of caspase-2 reduces spine pruning which leads to overall cognitive inflexibility, enhanced anxiety, and fear memory in *Casp2*^−/−^ mice [80]. Taken together, these findings demonstrate the importance of caspase-2 in cognition under physiologically normal conditions, but also render it a putative target in therapeutic treatment of neurodegenerative disorders (Fig. 4). To this end, interesting approaches have been proposed, such as inhibition of caspase-2 activation using TAT-fused PIDD1 and RAIDD-derived peptides to interfere with PIDDosome formation [91].

If the PIDDosome or an alternative complex containing RAIDD facilitates caspase-2 activation in neurons might depend on the upstream signal, yet both proteins were found to play a role in brain development, and mutations identified in patients were associated with mild forms of lissencephaly and intellectual disabilities [92–96].

The lissencephaly spectrum of diseases is characterized by a smoothened cerebral cortex surface due to reduced or absent folds (agyria to pachygyria). This malformation stems from defects in neuronal migration during brain development, which results in a thickened cortex. The symptoms range from developmental delay and seizures to intellectual disabilities [97]. Whole exome sequencing revealed mutations in RAIDD in lissencephaly patients from independent families of different ethnicities, and pedigree analysis suggests autosomal recessive inheritance [92]. Interestingly, exome sequencing and brain MRI data identified one of these RAIDD variants as potential founder mutation (Arg170His), as it is enriched in an isolated Finnish population with frequently occurring intellectual disabilities caused by a lissencephaly variant [98, 99].

How these phenotypes are caused is not fully resolved yet. Although these RAIDD variants were found to still bind to PIDD1 in a cellular system overexpressing the respective proteins, in vitro assays showed that these mutations disrupt interaction [92, 94]. Either way, the mutated variants of RAIDD failed to activate caspase-2. Hence, the authors suggest that the mild lissencephaly is caused by decreased cell death during cortical development rather than defective neuronal migration [92]. Intriguingly, another study on autosomal recessive intellectual disability identified a loss-of-function mutation in the PIDD1 death domain in two unrelated Pakistani families [95], and additional novel mutations in PIDD1 were identified and are currently characterized (John Vincent, personal communication, 2020), supporting the idea that PIDDosome formation is required for brain development or proper function. The relevance of RAIDD for cortical development was to some extent confirmed by the finding that *Raidd*^*−/−*^ mice exhibit mild megaencephaly, a form of lissencephaly [92]. Mice deficient for caspase-2 or PIDD1 have not been studied with regards to brain size or cognitive function and future work will have to show whether RAIDD mediates neuronal apoptosis in a PIDDosome-dependent context or exerts its function with alternative interactors.

## Conclusions

In summary, caspase-2, PIDD1, and RAIDD perform versatile functions in cell differentiation and tissue development. However, the downstream effectors mediating responses such as cell cycle exit or apoptosis are not fully understood and details on activating upstream signals are still unclear. As such, supernumerary centrosomes could potentially serve as activating cue for caspase-2 and the PIDDosome in several other polyploid cell types, including osteoclasts and myoblasts as well as cardiomyocytes during heart development [19, 57, 100]. Aside from activating signals, the roles of PIDD1 and RAIDD in these processes are clearly understudied. With the exception of liver polyploidization, trigger and mode of caspase-2 activation are still unclear in most tissues and cell types. Future studies will have to dissect if PIDD1 and RAIDD are solely responsible for caspase-2 activation or perform independent functions in other signaling complexes mediating the observed phenotypes in vivo. Moreover, it is unclear if all the phenotypes noted are entirely cell autonomous. Finally, these findings clearly render manipulation of caspase-2 activity promising for therapeutic intervention in fatty liver diseases but also neurodegenerative disorders and osteoporosis.
